# Melibiose Confers a Neuroprotection against Cerebral Ischemia/Reperfusion Injury by Ameliorating Autophagy Flux via Facilitation of TFEB Nuclear Translocation in Neurons

**DOI:** 10.3390/life11090948

**Published:** 2021-09-10

**Authors:** Zhiyuan Wu, Yongjie Zhang, Yuyuan Liu, Xuemei Chen, Zhiwen Huang, Xiaoming Zhao, Hongyun He, Yihao Deng

**Affiliations:** Department of Basic Medicine, Medical School, Kunming University of Science and Technology, Kunming 650500, China; yuan@stu.kust.edu.cn (Z.W.); 20192136033@stu.kust.edu.cn (Y.Z.); 20202136040@stu.kust.edu.cn (Y.L.); 20202136006@stu.kust.edu.cn (X.C.); 20202136033@stu.kust.edu.cn (Z.H.); 20190064@kust.edu.cn (X.Z.)

**Keywords:** ischemic stroke, melibiose, TFEB nuclear translocation, autophagy flux, neuroprotection

## Abstract

Autophagic/lysosomal dysfunction is a critical pathogenesis of neuronal injury after ischemic stroke. Trehalose has been validated to restore the impaired autophagy flux by boosting transcription factor EB (TFEB) nuclear translocation, but orally administrated trehalose can be greatly digested by intestinal trehalase before entering into brain. Melibiose (MEL), an analogue of trehalose, may thoroughly exert its pharmacological effects through oral administration due to absence of intestinal melibiase. The present study was to investigate whether melibiose could also confer a neuroprotection by the similar pharmacological mechanism as trehalose did after ischemic stroke. The rats were pretreated with melibiose for 7 days before middle cerebral artery occlusion (MCAO) surgery. Twenty-four hours following MCAO/reperfusion, the cytoplasmic and nuclear TFEB, and the proteins in autophagic/lysosomal pathway at the penumbra were detected by western blot and immunofluorescence, respectively. Meanwhile, the neurological deficit, neuron survival, and infarct volume were assessed to evaluate the therapeutic outcomes. The results showed that the neurological injury was significantly mitigated in MCAO+MEL group, compared with that in MCAO group. Meanwhile, nuclear TFEB expression in neurons at the penumbra was significantly promoted by melibiose. Moreover, melibiose treatment markedly enhanced autophagy flux, as reflected by the reinforced lysosomal capacity and reduced autophagic substrates. Furthermore, the melibiose-elicited neuroprotection was prominently counteracted by lysosomal inhibitor Bafilomycin A1 (Baf-A1). Contrarily, reinforcement of lysosomal capacity with EN6 further improved the neurological performance upon melibiose treatment. Our data suggests that melibiose-augmented neuroprotection may be achieved by ameliorating autophagy flux via facilitation of TFEB nuclear translocation in neurons after ischemic stroke.

## 1. Introduction

Ischemic stroke caused by cerebrovascular occlusion is a serious disease, resulting in millions of death throughout the world [1]. Moreover, a huge number of stroke survivors presents great economic and social burdens, due to the severe neurological deficits [2]. Pharmacological administration with the recombinant tissue plasminogen activator (rt-PA) is an effective thrombolysis therapy at the early stage of stroke, within 4.5 h after the insult, but this narrow therapeutic time window greatly limits its application, leading to only about 5% of the patients can benefit from this treatment [3]. Mechanical thrombectomy is another efficacious approach for cerebrovascular complete recanalization, but the sudden blood resupply may lead to more serious brain damage due to the ischemia/reperfusion injury [4]. Therefore, novel therapies are urgently needed to improve stroke treatment. Neuronal injury is the direct pathogenesis of brain damage after stroke [5]. Thus, understanding the pathological mechanism of neuronal injury may be a fundamental way to seek novel clues for stroke treatment.

Autophagy is extensively involved in the pathophysiological processes of cerebral ischemia. It is a metabolic machinery by which presenting aging proteins, damaged organelles, toxic proteins, and waste substrates to lysosomes for degradation [6]. Autophagy contains several processes including autophagy activation, formation of autophagosomes, fusion of autophagosomes with lysosomes, degradation of autophagic cargoes in autolysosomes. A consecutive state of these processes is termed “autophagy flux” [7]. The roles that autophagy played in the pathological processes are continuously controversial, but it is widely accepted that an excessive autophagy aggravates neurological injury, whereas a modest autophagy benefits neuroprotection after cerebral stroke [8]. However, it is elusive to determine what degree of autophagy can be considered appropriate or undue. Recent studies showed that a massive accumulation of autophagic substrates was accompanied with lysosomal incapacitation in neurons at the penumbra, indicating an impairment of autophagy flux was generated 24 h after ischemic stroke [9]. Meanwhile, the neurological damage was deteriorated at this stage, implying the autophagic/lysosomal dysfunction might be an important pathogeny of the neural injury after stroke [10]. Furthermore, reinforcing lysosomal capacity with cyclosporine A could ameliorate the autophagy flux and prominently alleviated the neuronal injury [11]. Contrarily, inhibition of lysosomal function with Bafilomycin A1 (Baf-A1) further aggravated the autophagic/lysosomal dysfunction and subsequently worsened the poststroke damage [12]. This indicated that the lysosomal inefficiency was the major cause of autophagic/lysosomal dysfunction after ischemic stroke. Thus, reinforcing lysosomal function might be an effective way to restore the impaired autophagy flux after cerebral stroke [10]. Meanwhile, studies suggested that facilitation of transcription factor EB (TFEB) nuclear translocation could alleviate the autophagic/lysosomal dysfunction by promotion of lysosome biosynthesis [13]. Accordingly, we asked whether the lysosomal dysfunction could be restored by promotion of lysosomal biosynthesis after cerebral ischemia [10,13].

The lysosome generation is strongly regulated by TFEB nuclear translocation, which is determined by its phosphorylation level [14]. After dephosphorylation, TFEB rapidly entered into the nucleus to activate the “coordinated lysosomal expression and regulation (CLEAR)” signaling, and in turn up-regulated the transcriptions of lysosomal genes to promote lysosomal biosynthesis [15]. When TFEB was phosphorylated, its activity was deactivated by binding to the adhesive protein of 14-3-3 within cytoplasm [16,17]. Accordingly, reduction of TFEB phosphorylation was an available way to boost its nuclear translocation. The mechanistic target of rapamycin kinase complex 1 (mTORC1) and protein kinase C (PKC) have been identified to be the main kinases for TFEB phosphorylation, but they simultaneously regulated multiple signaling, such as inflammation, oxidative stress, apoptosis, proliferation, differentiation, etc. [14,18,19]. Therefore, changing their activities was not the preferred approach to alter TFEB phosphorylation level, due to the fact that they likely triggered off-target concomitant effects.

Trehalose, a natural disaccharide primarily extracted from *Selaginella tamariscina*, has been verified to be a neuroprotective agent for treatment of center nervous system (CNS) diseases, such as Alzheimer’s disease and Parkinson’s disease [18,19]. In these investigations, trehalose was investigated to possess a bioactive property to promote lysosomal biosynthesis by boosting TFEB nuclear translocation [20]. However, orally administrated trehalose could be greatly digested by intestinal trehalase before entering into the brain, resulting in its pharmacological ineffectiveness [21,22,23]. Melibiose (MEL), an analogue of trehalose, might be maintained with a high therapeutic concentration in the brain by oral administration due to absence of intestinal melibiase [24]. Moreover, a study has indicated that melibiose could cross the blood-brain barrier (BBB) [25]. Thus, an oral medication of melibiose could be conveniently provided for treatment of ischemic stroke in the areas where healthcare was poorly developed. However, whether trehalose could confer a neuroprotection against cerebral ischemia/reperfusion injury remained to be explored. Besides, whether its therapeutic effects were exerted by modulating autophagic/lysosomal signaling was to be investigated in this study. If a neuroprotection against cerebral stroke was elicited by the same pharmacological mechanism as trehalose did, melibiose was to be exploited as a novel neuroprotective agent for treatment of ischemic stroke.

## 2. Materials and Methods

### 2.1. Experimental Animals

Adult male Sprague-Dawley rats, 250–280 g at the time of surgery, were purchased from Hunan SJA Laboratory Animal Co., Ltd., (license number: SCXK (xiang) 2019-0004, Changsha, Hunan, China), and housed 6 to 8 per cage under standard housing conditions with free access to food and water. All procedures and treatments were conducted following the ethical regulations set by the Animal Experimentation Committee of Kunming University of Science and Technology.

### 2.2. Cerebroventricular Cannulation for Intracerebroventricular Injection

Rats were anesthetized with 10% chloral hydrate (360 mg/Kg, Sangon Biotech, A600288, Shanghai, China) and were placed in a stereotaxic apparatus (RWD life science, 680001, Shenzhen, China) for implantation of the cannula into the brain lateral ventricles according to the coordination’s of AP = −0.9 mm, DV = +1.8 mm, ML = −3.5 mm. (AP, anterior from the bregma; DV, distance below the surface of the brain; ML, lateral from the midline).

### 2.3. Drug Administration

To investigate the effects of melibiose (Huaxia Reagent, P639485, Chengdu, China) on the pathological damage after ischemic stroke, MEL (24 mg/Kg/d, i.p.) was pre-treated once daily for 7 days before MCAO surgery and given an additional administration after onset of MCAO/reperfusion. The administration dose in rats was determined by calculating an equivalent dose per unit weight to that used in mice [25]. To uncover the effect of MEL on lysosomal capacity, the lysosomal activity was altered by Bafilomycin A1 (Baf-A1, Selleck, S1413, Shanghai, China) and EN6 (Selleck, S6650, Shanghai, China) upon MEL treatment, respectively. Baf-A1 (2 μg/kg/d) or EN6 (10 μg/kg/d) was injected into the lateral ventricle with a micropump (1 μL/min, RWD life science, R452, Shenzhen, China) [26,27] once daily for 7 days of pre-treatment and was given an additional administration on the day of MCAO/reperfusion surgery.

### 2.4. Rat Model of Ischemic Stroke Was Prepared by MCAO/Reperfusion

The model of ischemic stroke in rat was prepared by middle cerebral artery occlusion (MCAO), as described in our previous study [28]. Briefly, rats were anesthetized with 10% chloral hydrate (Sangon Biotech, A600288, 360 mg/kg, Shanghai, China) and fixed on an operating table. After isolation of the target arteries, a MCAO filament (Cinontech, 2636-A5, Beijing, China) was inserted into the left internal carotid artery through an incision on the external carotid artery. After that, the filament was gently advanced into the middle cerebral artery for blockage of the blood flow. After 1.5 h of occlusion, the filament was withdrawn to allow reperfusion. During and after MCAO operation, the rats were placed on an animal electrothermal pad (Rainbow, TG104-X32, Chengdu, China) to maintain their body temperature at about 37 °C. Approximately 2 h after the MCAO/reperfusion, the rats were removed into rat cages when they regained consciousness from anesthesia. The rats were sacrificed 24 h after the MCAO/reperfusion. The rats in the sham group received all the operational procedures, except for insertion with a filament.

### 2.5. Neurological Deficit Score Was Assessed to Evaluate the Pharmacological Effects of MEL

The neurological injury was evaluated by the modified Neurological Severity Score (mNSS) test, according to the reported study [29]. Four items were assessed in the mNSS test, including sensory function, reflex evaluation, balance ability, and motor function. The neurological score is on a scale of 0 to 18. A higher score indicated more severe neurological deficit.

### 2.6. The Brain Infarct Volume Was Measured by TTC Staining

Twenty-four hours after MCAO/reperfusion, the brains were isolated and immediately frozen at −20 °C for 20 min, and then coronally cut into 2mm-thickness sections. The sections were incubated in a 2% solution of 2,3,5-triphenyl tetrazolium chloride (TTC; Solarbio, G3005, Beijing, China) at 37 °C for 20 min and fixed with 4% para-formaldehyde for 30 min. The staining images were photographed with a digital camera (Canon, EOS 850D, Tokyo Metropolitan, Japan). The normal tissues were red and the infarcted areas were shown pale by TTC staining. The infarct volume was measured and calculated by a Adobe Photoshop 2020 (Adobe Systems, San Jose, CA, USA) software. The result was expressed by the infarct ratio (%) = infarct volume /the volume of the ipsilateral hemisphere × 100%.

### 2.7. Neuronal Survival Was Detected by Nissl Staining

The brain sections obtained above were first fixed on the slides. After xylene dewaxing and gradient alcohol hydration, the brain sections were stained with a Nissl staining solution (Sangon Biotech, E607316, Shanghai, China) for 5 min at room temperature. After washing with distilled water, they were differentiated with 95% alcohol until the Nissl substances were turned bluish-violet. Subsequently, they were treated with pure alcohol and xylol. Finally, the sections were sealed with neutral gum. The Nissl staining were observed and captured with a fluorescence microscope (Nikon, ECLIPSE Ci-L, Tokyo Metropolitan, Japan) under high magnification (20×). The Nissl bodies were counted in 10 non-overlapping fields in each section, and 5 sections were randomly selected from each detected sample. The result was represented by the number of Nissl bodies per field.

### 2.8. Neuron Loss Was Detected by Fluoro-Jade C Staining

To evaluate the neuron loss at the penumbra after MCAO/reperfusion, the brain sections were stained by a Fluoro-Jade C (FJC) staining kit (Biosensis, TR-100-FJT, Thebarton, Australia), according to the instructions provided by the manufacturer. The sections were first immersed with a NaOH/ethanol (1%/80%) solution, and they were sequentially dealt with 70% ethanol solution and distilled water, respectively. After that, they were incubated with 0.06% potassium permanganate solution for background removal. After washing, the sections were stained with the FJC solution for 10 min in the dark and were cleaned with xylene. The staining images were captured with a fluorescence microscope (Nikon, ECLIPSE Ci-L, Japan) under high magnification (20×). The number of FJC-positive cells in 10 non-overlapping fields was counted in each section, and 5 sections had to be randomly selected for counting. The result was expressed by the number of FJC-positive cells per field.

### 2.9. Western Blot

The brain tissue from the penumbra was obtained 24 h after MCAO/reperfusion. The protein was extracted by a Nuclear and Cytoplasmic Protein Extraction Kit (Beyotime Biotechnology, P0028, Shanghai, China). BCA protein assay kit (Beyotime Biotechnology, P0010) was used to measure the total protein concentration. Proteins (15 mg) were separated by 10–12% SDS-polyacrylamide gel electrophoresis and blotted onto PVDF membranes (Millipore, IPVH00010, Darmstadt, Germany). The membranes were then blocked with 10% skimmed milk (Beyotime Biotechnology, P0216-300g) in Tris-buffered saline (Solarbio, T8060, Beijing, China) containing 0.1% Tween 20 (Solarbio, T8220, Beijing, China) (TBST) at room temperature for 1 h. After blocking, the membranes were washed with TBST and then incubated with rabbit primary antibodies against rat LC3 (1:1000, Sigma, L7543, Darmstadt, Germany), SQSTM-1 (1:4000, Proteintech, 18420-1-AP, Wuhan, China), cathepsin D (1:5000, Proteintech, 66534-1-AP), LAMP-2 (1:1000, Novusbio, NBP2-22217, Centennial, CO, USA), TFEB (1:1000, Proteintech, 13372-1-AP), Histone H3 (1:1000, Affinity Biosciences, AF0863, Cincinnati, OH, USA), and GAPDH (1:20000, Proteintech, 10494-1-AP), and mouse primary antibodies against rat Beclin-1 (1:500, ABclonal, A10101, Wuhan, China), cathepsin B (1:500, Santa Cruz Bio-technology, 66534-1-AP, Dallas, TX, USA), and β-Actin (1:5000, Affinity, T0022) at 4 °C overnight. The immune complexes were detected by horseradish peroxidase conjugated secondary antibodies at room temperature for 2 h. After that, the membranes were washed and submerged in Immobilon Western Chemiluminescent HRP substrate (Millipore, WBKLS0500). The protein signals were detected by X-ray film. Band patterns were analyzed with Image J software and normalized to the loading controls.

### 2.10. Immunofluorescence

Animals were transcardially perfused with PBS (pH 7.4) followed by 4% para-formaldehyde (vol:vol). Brains were immersed in 4% paraformaldehyde, immersed in 20% and 30% sucrose (Solarbio, S8271, Beijing, China) until they were observed to sink to the bottom. After that, the brains were sliced into sections (20 μm of thickness) with a freezing microtome (SLEE, MEV, Mainz, Germany. The sections were washed and permeabilized for 1 h with 0.3% Triton X-100 in PBS. Afterward, the sections were blocked with 10% BSA (Beyotime Biotechnology, ST025, Shanghai, China) for 1 h at room temperature. After then, they were incubated with primary antibodies against SQSTM-1 (1:100, Proteintech, 18420-1-AP, Wuhan, China), cathepsin D (1:200, Proteintech, 66534-1-AP), TFEB (1:100, Proteintech, 13372-1-AP), and NeuN (1:1000, Abcam, ab104224, Cambridge, UK) overnight at 4 °C. The sections were then washed with PBS and labeled with Alexa Fluor 488- and Alexa Fluor 555-conjugated secondary antibodies (1:800, Jackson, 151181&150641, West Grove, PA, USA) in the dark for 1 h at room temperature. Thereafter, the sections were washed and counterstained with DAPI (Sigma, D9542, Darmstadt, Germany) for nuclear staining. Under high magnification (40×), the staining images were observed and captured by a fluorescence microscope (Nikon, ECLIPSE Ci-L, Japan). The number of positive and the total number of cells were counted in 10 non-overlapping fields in each section, and 5 sections were randomly selected from each sample. The result was expressed by the percentage (%) of the reaction-positive cells/the total number of cells.

### 2.11. Statistical Analysis

For statistical analysis, GraphPad Prism version 9 (San Diego, CA, USA) was used. Western blot quantification was performed with ChemiDoc software. Data was analyzed with one-way of variance (ANOVA) followed by the Dunnett test. Data are ex-pressed as means ± SEM of 6 independent samples. *p* < 0.05 was considered statistically significant.

## 3. Results

### 3.1. MEL Prominently Ameliorated the Autophagy Flux at the Penumbra after Ischemic Stroke

**Figure 1:**
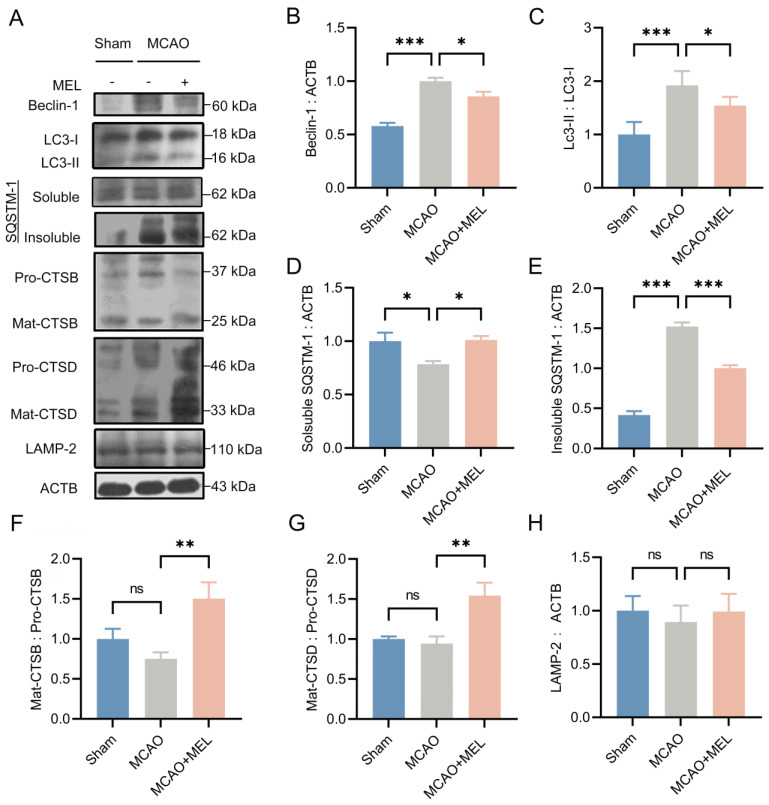
MEL greatly restored the autophagic/lysosomal dysfunction after ischemic stroke. MEL treatment prominently decreased the ratio of LC3-II/LC3-I (**A**,**C**) and Beclin-1 (**B**) expression at the ischemic penumbra. Meanwhile, MEL significantly reduced the autophagic substrate of insoluble SQSTM-1(**D,E**). This decreased SQSTM-1 might be elicited by the reinforced lysosomal activation (**F**) and its degradative activities of CTSB (**G**) and CTSD (**H**). MCAO, middle cerebral artery occlusion. MEL, melibiose. CTSB, cathepsin B. CTSD, cathepsin D. n = 6, * *p* < 0.05, ** *p* < 0.01, *** *p* < 0.001, ns indicates that the data are not statistically different.

Autophagic/lysosomal dysfunction has been investigated to be an important cause of neuronal injury after ischemic stroke [30]. To investigate whether MEL could restore this dysfunction, the key proteins in the autophagy-lysosomal pathway were detected by western blot 24 h after middle cerebral artery occlusion (MCAO)/reperfusion (Figure 1A). The result showed that an impaired autophagy flux was created by the MCAO/reperfusion, as reflected by the increased autophagic accumulation in MCAO group, compared with that in sham group (Figure 1E). The autophagic activities of LC3-II and Beclin1 were significantly suppressed by melibiose treatment in MCAO+MEL group, compared with those in MCAO group (Figure 1C). This implied the generation of autophagic cargoes might be reduced by MEL. Meanwhile, the lysosomal capacity was markedly enhanced by MEL treatment, as indicated by the promoted lysosomal expressions of cathepsin B (Figure 1F) and cathepsin D (Figure 1G), accompanying with attenuated autophagic substrates of LC3-II (Figure 1C) and insoluble SQSTM-1 (Figure 1E). These data suggested that MEL treatment significantly ameliorated the autophagic/lysosomal signaling after ischemic stroke.

### 3.2. The MEL-Ameliorated Autophagy Flux Might Be Achieved by Induction of TFEB Nuclear Translocation in Neurons

**Figure 2:**
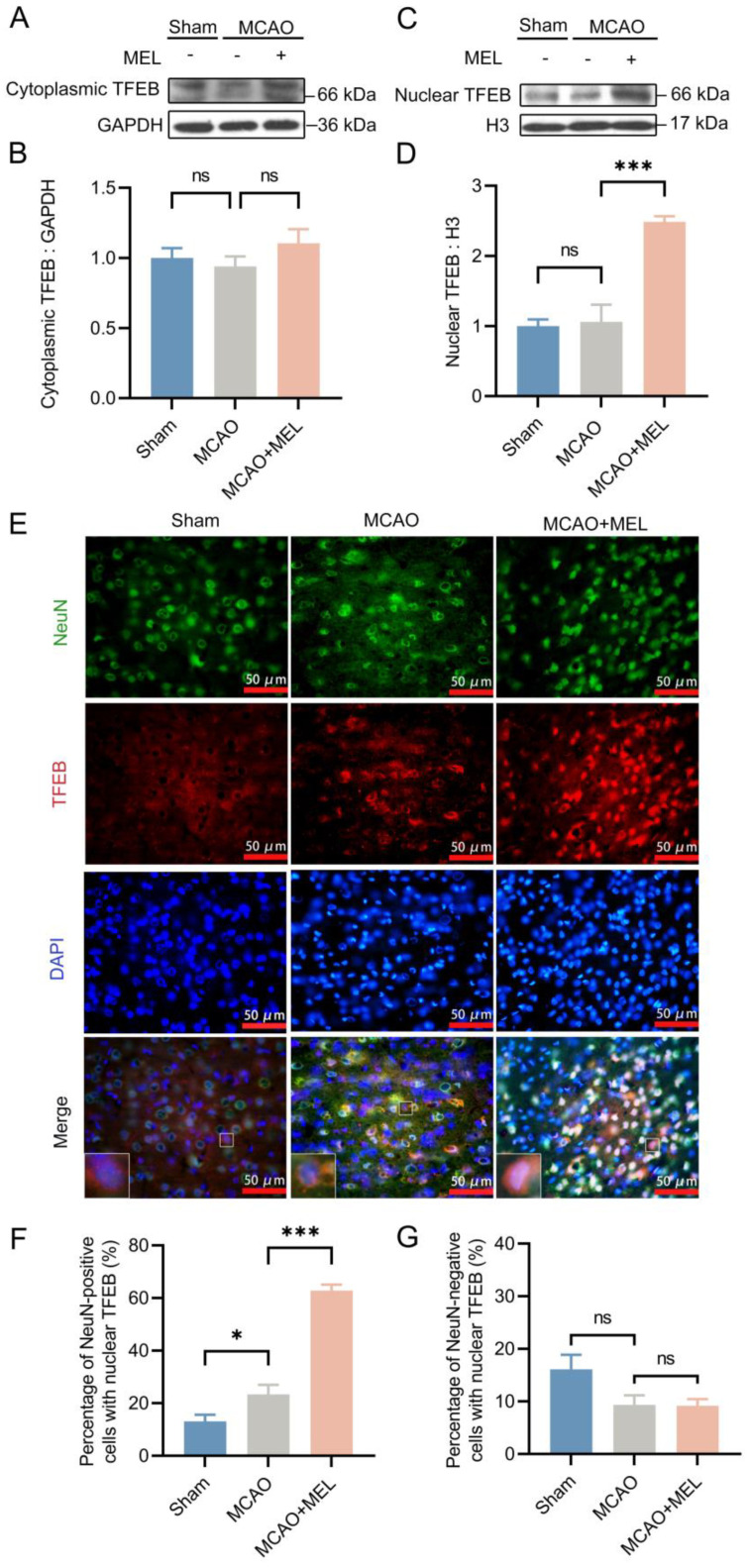
MEL boosted TFEB nuclear translocation in the neurons at the penumbra after ischemic stroke. Western blot showed that TFEB expression in the nucleus was prominently increased by MEL treatment after MCAO/reperfusion (**A**–**D**). Moreover, double immunofluorescence indicated that the TFEB nuclear translocation was mainly displayed in the neurons at the penumbra (**E**–**G**). n = 6, * *p* < 0.05, *** *p* < 0.001, ns *p* > 0.05.

To observe whether the MEL-ameliorated autophagy flux was exerted by boosting TFEB nuclear translocation, the TFEB expressions in cytoplasm and nucleus were detected, respectively. Western blot (Figure 2A,C) showed that the nuclear TFEB expression were obviously promoted by melibiose treatment in MCAO+MEL group, compared with that in MCAO group (Figure 2D). This suggested that MEL possessed the pharmacological efficacy to facilitate TFEB nuclear translocation after ischemic stroke. Moreover, double immunofluorescence showed that the number of NeuN-positive cells co-labeled with TFEB and DAPI was significantly increased by MEL treatment (Figure 2E,F). However, the number of cells colocalized with TFEB and DAPI in NeuN-negative cells was statistically similar among groups (Figure 2G). This indicated that MEL-facilitated TFEB nuclear translocation was mainly occurred in neurons at the penumbra.

### 3.3. Enhancing the Lysosomal Capacity Was the Main Contribution of MEL-Boosted TFEB Nuclear Translocation

**Figure 3:**
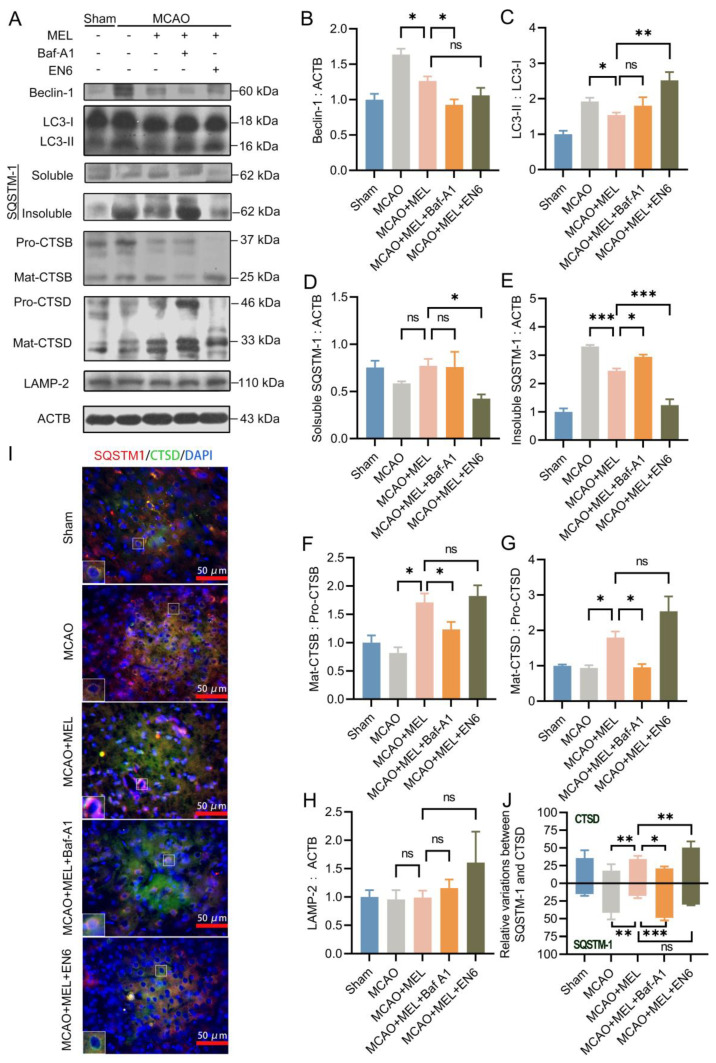
The MEL-ameliorated autophagy flux was achieved mainly by reinforcing the lysosomal capacity after MCAO/reperfusion. MEL treatment significantly inhibited autophagic activity (**A**–**C**), which likely decreased the generation of autophagic cargoes (**D**,**E**). Meanwhile, MEL markedly reinforcing the lysosomal activities of CTSB (**F**), CTSD (**G**), and LAMP2 (**H**), which necessarily promoted the degradation of autophagic substrates. Immunofluorescence (**I**) also indicated that MEL could promote CTSD expression and simultaneously attenuated the autophagic substrate of SQSTM-1 (**J**). Thus, the autophagy flux was prominently ameliorated by MEL treatment. However, the effect of MEL to ameliorate autophagic/lysosomal signaling was greatly counteracted by lysosomal inhibitor Baf-A1. Conversely, MEL-improved autophagy flux was further facilitated by EN6. n = 6, * *p* < 0.05, ** *p* < 0.01, *** *p* < 0.001, ns indicates that the data are not statistically different.

TFEB was the master regulator of lysosomal biosynthesis [13], in order to investigate whether MEL-boosted TFEB nuclear translocation could alter the lysosomal capacity, the lysosomal inhibitor Baf-A1 and agonist EN6 were administrated upon MEL treatment, respectively. The result (Figure 3A) showed that MEL-enhanced autophagic/lysosomal pathway was greatly canceled by Baf-A1, as indicated by the decreased lysosomal activities of cathepsin B (Figure 3F) and cathepsin D (Figure 3G), accompanying with increased insoluble SQSTM-1 (Figure 3E). Conversely, lysosomal agonist EN6 further facilitated the MEL-boosted autophagy flux (Figure 3C). Furthermore, immunofluorescence (Figure 3I) indicated that MEL treatment significantly promoted lysosomal activity of cathepsin D, whereas it decreased the autophagic substrate of insoluble SQSTM-1. However, the effect of MEL to enhance the lysosomal capacity was prominently counteracted by Baf-A1 (Figure 3J). These outcomes indicated that MEL-ameliorated autophagy flux was achieved mainly by enhancement of lysosomal function.

### 3.4. MEL Dramatically Promoted Neuron Survival at the Penumbra

**Figure 4:**
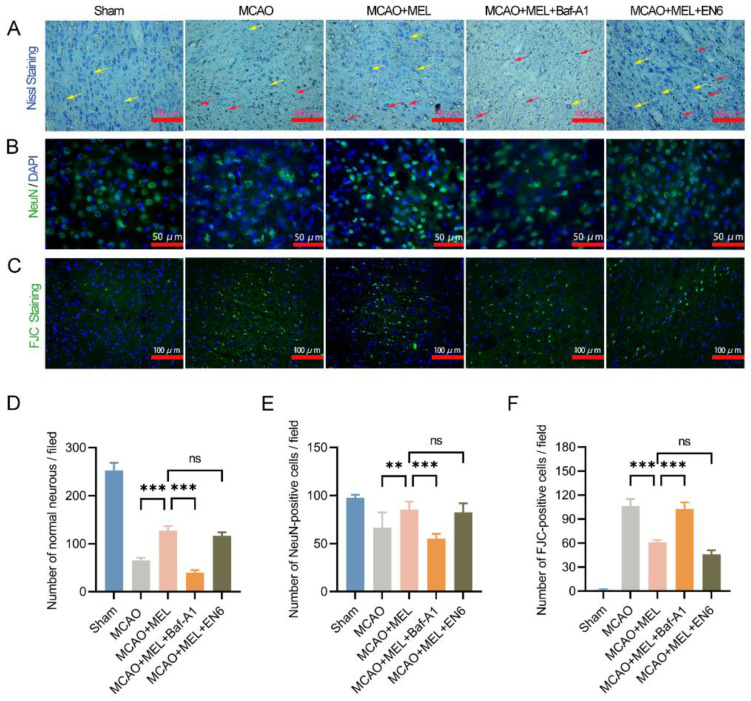
MEL treatment attenuated neuron loss at the penumbra after MCAO/reperfusion. MEL significantly promoted neuron survival, as showed by increased number of Nissl bodies (**A**,**D**) and NeuN-positive cells (**B**,**E**) at the penumbra. Meanwhile, the neuron loss detected by FJC staining was obviously decreased by MEL treatment (**C**,**F**). However, the effect of MEL to promote neuron survival was greatly canceled by lysosomal inhibitor Baf-A1. The yellow arrows indicated viable neurons and the red arrows showed pyknotic neurons. n = 6, ** *p* < 0.01, *** *p* < 0.001, ns indicates that the data are not statistically different.

Nissl staining (Figure 4A), immunofluorescence (Figure 4B) and FJC staining (Figure 4C) were, respectively, employed to evaluate neuron survival at the penumbra 24 h after ischemic stroke. Our data showed that MEL treatment significantly promoted the number of Nissl bodies (Figure 4D) and NeuN-positive cells (Figure 4E) at the penumbra in MCAO+MEL group (Figure 4F), compared with those in MCAO group. Moreover, the number of FJC-positive cells could also be obviously reduced by MEL. However, the pharmacological effect of MEL to promote neuron survival was greatly counteracted by lysosomal inhibitor Baf-A1. Contrarily, lysosomal activator EN6 could further alleviated the neuronal injury upon MEL treatment. These data suggested that MEL-promoted neuron survival was elicited mainly by restoring the lysosomal inefficacy after ischemic stroke.

### 3.5. MEL Dramatically Attenuated Brain Infarct Volume after MCAO/Reperfusion

**Figure 5:**
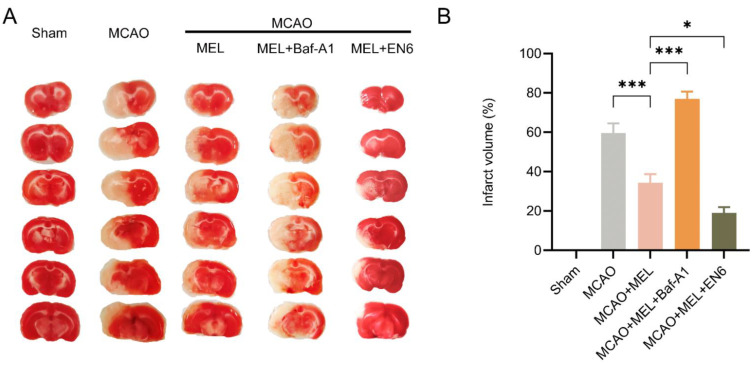
MEL treatment prominently attenuated the infarct volume after ischemic stroke. MEL treatment could significantly reduce the infarct size after ischemic stroke (**A**). However, this effect was greatly abolished by lysosomal inhibitor Baf-A1 (**B**). n = 6, * *p* < 0.05, *** *p* < 0.001.

To investigate the effect of MEL treatment on brain damage, the infarct volume was measured by TTC staining 24 h after ischemic stroke (Figure 5A). The result showed that the infarction size was prominently attenuated by melibiose in MCAO+MEL group, compared with that in MCAO group. The infarct volume was further enlarged by lysosomal inhibitor Baf-A1, but it was contrarily decreased by lysosomal activator EN6 upon MEL treatment (Figure 5B). These data indicated that MEL could effectively alleviate the ischemic brain injury, and this pharmacological effect might be exerted by enhancement of lysosomal function.

### 3.6. MEL Markedly Alleviated the Neurological Deficit and Reduced Animal Mortality after Ischemic Stroke

**Figure 6:**
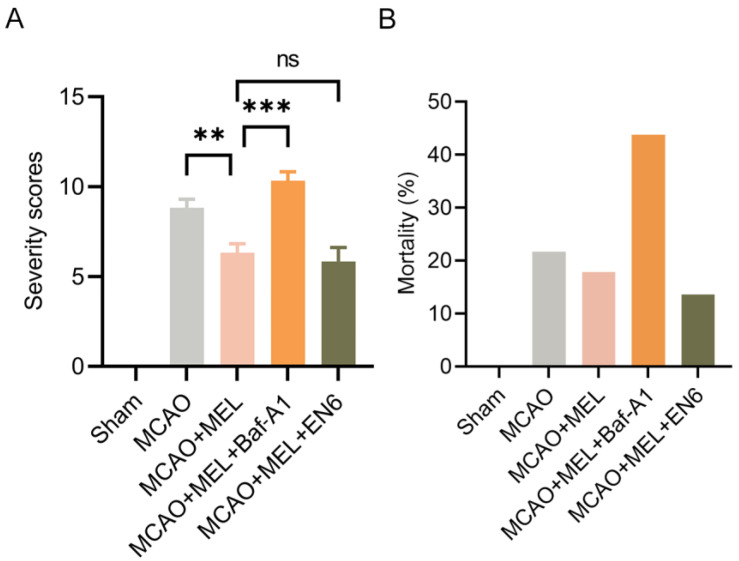
Neuroprotective effects of MEL was evaluated by mNSS test and animal mortality. MEL treatment effectively alleviated the neurological deficit (**A**) and animal mortality (**B**) after the MCAO/reperfusion. However, MEL-elicited neuroprotection could be greatly counteracted by lysosomal inhibitor Baf-A1. n = 6, ** *p* < 0.01, *** *p* < 0.001, ns indicates that the data are not statistically different.

The modified neurological severity scores (mNSS) was used to evaluate neurofunction 24 h after MCAO/reperfusion (Figure 6A). The result showed that the neurological deficit was significantly attenuated by melibiose treatment in MCAO+MEL group, compared with that in MCAO group. However, this protective effect was obviously counteracted by lysosomal inhibitor Baf-A1. Conversely, the lysosomal activator EN6 played a synergistic roles with MEL treatment in attenuation of neurological deficiency. Moreover, the animal mortality (Figure 6B) could be effectively reduced by melibiose in MCAO+MEL group, compared with that in MCAO group. However, this pharmacological effect was greatly counteracted by lysosomal inhibitor Bafilomycin A1 (Baf-A1).

## 4. Discussion

The exact roles that autophagy played in the pathology of cerebral stroke were continuously debated [31]. In fact, autophagy contained several consecutive processes, including autophagy initiation, autophagosomes maturation, autolysosomes formation by fusion of autophagosomes with lysosomes, and autophagic degradation in autolysosomes [32,33]. A consecutive and integral state of these processes was termed autophagy flux. Numerous reported investigations determined the roles of autophagy in ischemic stroke only by evaluation of autophagic activity [34,35]. However, they seldom concerned the dynamic variations between each process of autophagy flux and pathological development of cerebral ischemia. This might be the reason why controversial and even opposite conclusions were drawn among previous studies [36,37]. Accordingly, the each step in the autophagic/lysosomal pathway was entirely monitored to uncover the pharmacological mechanism of melibiose in our study.

Several observations have indicated that autophagic/lysosomal dysfunction mainly caused by lysosomal inefficiency was an important pathogenesis of neuronal injury after acute stroke [10,38,39,40]. Accordingly, promotion of lysosomal biosynthesis might be an essential way to restore the impairment of autophagy flux [13]. Lysosome generation was positively regulated by TFEB nuclear translocation, which was determined by its phosphorylation state [41]. A higher level of TFEB phosphorylation indicated its more inactivation with cytoplasmic retention, whereas a higher level of TFEB dephosphorylation implied its more activation with nuclear translocation [42]. Thus, reduction of TFEB phosphorylation was an effective method to boost its nuclear translocation. The mTORC1 was considered to be a major kinase for TFEB phosphorylation [43]. Theoretically, the level of TFEB phosphorylation could be decreased by mTORC1 inhibition to facilitate its nuclear translocation, which, in turn, promoted lysosomal biosynthesis to ameliorate autophagy flux [14]. However, studies indicated that lysosomal biosynthesis was independent on mTORC1. Recent studies uncovered that TFEB nuclear translocation could be facilitated by trehalose [20,22]. However, trehalose could be greatly digested by intestinal trehalose, leading to that it hardly entered into the brain through oral administration. Melibiose, an analogue of trehalose, might be maintained with an effective therapeutic concentration in the brain to exert its pharmacological effects by oral administration, due to absence of intestinal melibiase [44]. We, therefore, discussed whether MEL could also elicit a neuroprotection against ischemic stroke by boosting TFEB nuclear translocation.

The result demonstrated that the neurological injury was significantly mitigated by MEL treatment, as indicated by the attenuated infarct volume, reduced neurological deficits, promoted neuron survival, and decreased animal mortality in the MCAO+MEL group, compared with those in MCAO group 24 h after MCAO/reperfusion. The TFEB expression in nucleus was obviously promoted by MEL treatment, illustrating TFEB nuclear translocation was boosted. To further investigate the effects of melibiose-facilitated TFEB nuclear translocation on autophagy flux, the key proteins in the autophagic-lysosomal pathway were detected. Western blot showed that the impaired autophagy flux could be effectively restored by MEL, as reflected by the promoted lysosomal expressions of CTSB and CTSD, accompanied by decreased autophagic substrates of insoluble SQSTM-1. These results suggested that the melibiose-induced neuroprotection was exerted by facilitating TFEB nuclear translocation, being similar to the effects exerted by trehalose [20]. Furthermore, reinforcement of lysosomal function with EN6 further enhanced autophagic/lysosomal signaling and played a synergistic role in the alleviation of neurological injury upon MEL treatment. However, the restored autophagy flux by MEL was greatly counteracted by inhibition of lysosomal capacity with Baf-A1, as shown by increased accumulation of autophagic wastes and decreased lysosomal expressions in the MCAO+MEL+Baf-A1 group, compared with those in the MCAO+MEL group. This indicated that the effect of MEL to ameliorate autophagy flux was achieved mainly by augment of lysosomal function after ischemic stroke.

This study preliminarily elucidated the neuroprotective mechanism of MEL to enhance autophagy flux by induction of TFEB nuclear translocation in neurons after ischemic stroke. The cytoplasmic retention or nuclear translocation of TFEB was determined by its phosphorylation level. There were multiple phosphorylation sites on TFEB, but which phosphorylated sites were responsible for cytoplasmic retention and which dephosphorylated sites contributed to nuclear translocation were widely unknown [13]. Therefore, the level of TFEB phosphorylation was not directly assessed to evaluate its nuclear translocation in this study. Instead, the TFEB nuclear translocation was distinguished by its cytoplasmic and nuclear expressions through western blot and subcellular localization with immunofluorescence, respectively. Our data indicated that MEL was not only able to boost TFEB nuclear translocation but also could augment the autophagic/lysosomal signaling in neurons at the penumbra after MCAO/reperfusion. We, therefore, concluded that melibiose-elicited neuroprotection was exerted by ameliorating autophagy flux via facilitation of TFEB nuclear translocation in neurons after ischemic stroke.

## Data Availability

Data can be made available from the corresponding author upon reasonable request.
